# Case Report: Ascending hope—urgent endovascular repair of aortic pseudoaneurysm

**DOI:** 10.3389/fcvm.2025.1532920

**Published:** 2025-09-03

**Authors:** Daniel Ronen, David Planer, Ronen Beeri, Amit Korach, Gabby Elbaz Greener, Eldad Rahamim

**Affiliations:** ^1^ Department of Cardiology, Hadassah Medical Center, Jerusalem, Israel; ^2^ Faculty of Medicine, Hebrew University of Jerusalem, Jerusalem, Israel; ^3^ Department of Cardiothoracic Surgery, Hadassah Medical Center, Jerusalem, Israel

**Keywords:** ascending aorta, pseudoaneurysm, endovascular, invasive cardiology, heart team

## Abstract

We report a case of an ascending aortic pseudoaneurysm that developed as a complication of coronary artery bypass graft surgery and was managed with percutaneous endovascular repair. A 59-year-old man presented 9 months after bypass surgery, with complaints of dysphagia and shortness of breath. Computed tomography and echocardiography demonstrated a pseudoaneurysm of the ascending aorta. The patient's overall condition put him at a very high perioperative risk, prohibiting surgical intervention. However, the patient deteriorated clinically and suffered cardiac arrest, requiring repeated resuscitation efforts. In need of an urgent solution, the heart team recommended the interventional cardiology team perform a percutaneous endovascular repair, which resulted in significant hemodynamic improvement. Patients' clinical improvement allowed the surgical team to conduct a sternotomy to remove substantial mediastinal blood clots and place a bovine patch on a residual aortic leak. After recovery, the patient was discharged home and at 1-year follow-up is doing well. This case demonstrates the feasibility of percutaneous endovascular treatment in medical emergencies of the ascending aorta, when standard surgical treatment carries unacceptable risk.

## Introduction

Pathologies of the aorta carry significant morbidity and mortality, with acute thoracic aortic syndromes considered a medical emergency (1). Anatomical classifications, such as Stanford and DeBakey, are of extreme importance, as the involvement of the ascending aorta carries both prognostic and therapeutic consequences. Endovascular repair of pathologies of the descending aorta and even the aortic arch has become the standard of care. In contrast, for pathologies of the ascending aorta, surgical repair is the gold standard (1). This may be attributed to anatomical limitations in stent design and deployment. These include the ascending aorta’s short length, large asymmetric curvature, the need to protect the aortic valve, and the coronary and innominate arteries (2). An aortic pseudoaneurysm is a rare aortic pathology in which all layers of the aortic wall are compromised. Blood collection is created outside the aortic wall and is surrounded only by connective tissue. This carries the risk of imminent life-threatening rupture but can also press on surrounding structures. Pseudoaneurysms typically develop after surgery, ulcer, dissection, trauma, or infections (3). Typically, minimal symptoms are expected; however, once detected, there is a need for urgent, invasive treatment (1, 4). Data from the International Registry of Acute Aortic Dissection shows that surgical intervention reduces in-hospital mortality to 16.1% compared to 52.1% on medical treatment alone (5). Unfortunately, the already high perioperative morbidity and mortality risk is further complicated in elderly patients or patients with significant comorbidities, including previous cardiovascular surgery (6). Indeed, surgery is declined for approximately 10%–14% of patients due to high perioperative risk (7); this rate increases up to 31.9% in elderly patients aged >70 years (5). This lack of treatment options creates an unmet need for minimally invasive yet life-saving solutions. To date, most experience in endovascular repair of the ascending aorta comes from case reports using stents designed for the descending aorta (8). Other percutaneous methods reported include the placement of occluder devices (9–13) and the endo-Bentall procedure (14).

Recently, the prospective first-in-human, Ascending Aorta Repair: Improving Surgical Excellence (ARISE) (15) study showed promising results using a stent design dedicated to the ascending aorta.

Here, we report a case of a patient with post-coronary artery bypass grafting (CABG) surgery ascending aortic pseudoaneurysms treated endovascularly due to extreme surgical risk.

## Case description

A 59-year-old white man with a history of hypertension, diabetes mellitus, hyperlipidemia, and obesity underwent CABG surgery due to complex ischemic heart disease at the referring hospital 9 months before his transfer to our institution. CABG was performed on-pump with cannulation in the right atrium and the distal ascending aorta. The cardioplegia canula was placed 2 cm above the right coronary artery (RCA) ostium. The left internal mammary artery (LIMA) was grafted to the left anterior descending artery (LAD). A saphenous vein (SVG) was grafted to the first obtuse marginal and diagonal artery, and another SVG was grafted to the distal right coronary artery (RCA).

During his initial stay after CABG, the patient experienced surgical site bleeding, which was managed conservatively, and post-pericardiotomy syndrome, for which he received glucocorticosteroids. Both situations resolved and the patient was discharged home 10 days postoperatively. The patient was readmitted 14 days after initial discharge due to sternal dehiscence and suspected mediastinitis. A second sternotomy was performed with removal of a retrosternal collection and sternal rewiring was performed. Although bone and blood cultures taken during the hospital stay were negative, the patient was treated with prophylactic antibiotics. The patient was discharged home; however, he was uncompliant with his antibiotic treatment after discharge. Follow-up confirmed gradual improvement and return to activity. Initial transthoracic echocardiography (TTE) shortly after the procedure demonstrated preserved left ventricular (LV) systolic function with normal aortic morphology. At 5 months after discharge, a transesophageal echocardiography (TEE) also showed normal aortic morphology. At 7 months after discharge, a TEE did note a mildly dilated ascending aorta (37 mm) (Supplementary Figure S1).

**Figure 1:**
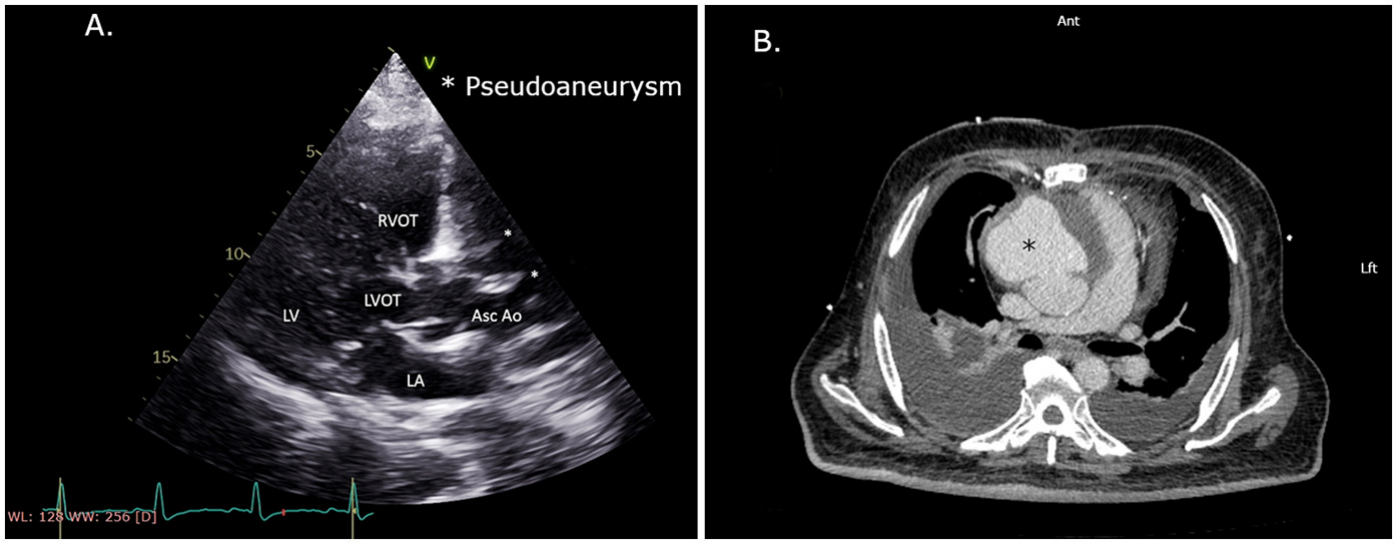
**(A)** Echocardiography. PLAX view with a decrease in left ventricular function and suspected pseudoaneurysm of the ascending aorta. **(B)** CTA scan with an anterior ascending aorta pseudoaneurysm showing a mass effect on the heart and pulmonary arteries. CTA, computed tomography angiography; PLAX, parasternal long-axis.

At 9 months after initial surgery, the patient became symptomatic and developed dysphagia and shortness of breath and was admitted to the same referring hospital. At this time, his TTE demonstrated moderate reduction in left ventricular systolic function with a suspected pseudoaneurysm of the ascending aorta (Figure 1A). Computed tomography angiography (CTA) confirmed the diagnosis with an anterior ascending aortic pseudoaneurysm measuring 11.5 cm over 8.5 cm, originating 2 cm above the RCA ostium (Figure 1B). The CTA also indicated that only the LIMA to the LAD graft was patent, while both SVG grafts and the native RCA were occluded.

The patient's hemodynamic instability and the two recent sternotomies with postoperative complications led the referring hospital and additional tertiary hospitals to deem the surgical risk prohibitive. Indeed, later during his stay at the referring hospital, the patient suffered a pulseless electrical activity (PEA) cardiac arrest event with short resuscitation and return of spontaneous circulation (ROSC). Thereafter, the patient was urgently transferred to our facility, 9 days after hospitalization.

Upon arrival at our center, the patient was pale and mildly diaphoretic. His vital signs were stable, with a blood pressure of 122/66 mmHg. The patient was tachypneic with an O_2_ saturation of 97% on minimal oxygen supplementation. On physical examination, a systolic murmur with no radiation was heard over the upper left sternal border. His lungs were clear by auscultation. Examination of his chest wall showed a well-healing sternal scar. No abdominal pulsations were palpated. Lower limbs showed poorly healed graft wounds and bilateral symmetric edema. Electrocardiography revealed a normal sinus rhythm, with no ST segment deviations and no conduction abnormalities or arrhythmias.

Supportive care and IV antibiotics were initiated. A few minutes after his arrival, the patient deteriorated with increased weakness and shortness of breath, followed by a sudden loss of consciousness, leading to PEA cardiac arrest. Full resuscitation was performed, resulting in return of spontaneous circulation (ROSC) within a few minutes. A second PEA cardiac arrest occurred 20 min later and resuscitation was performed again. The patient was sedated and intubated, followed by ROSC. Laboratory workup demonstrated mild microcytic hypochromic anemia (Hb 9.6 g/dl) with normal white blood cell and platelet counts. Kidney function and electrolytes were within the normal range. The N-terminal-pro-B-type natriuretic peptide (NT-proBNP) level was elevated (2,792 pg/ml), and the lactic acid level was elevated (38.9 mg/dl). C-reactive protein was mildly elevated (2.44 mg/dl), and blood cultures from the referral hospital were reported to be negative. Acute ischemia was ruled out as the patient did not suffer any chest pain, his ECG showed no ischemic changes, and high-sensitivity Troponin-I was negative. In addition, acute SVG graft occlusion was unlikely as it was detected at the referring hospital 9 days before he arrived at our center.

A heart team of interventional cardiologists, cardiothoracic surgeons, and imaging cardiologists was urgently assembled. CTA and TTE imaging of the patient's aortic anatomy suggested an adequate landing zone above the coronary arteries and sufficient space to spare the innominate artery and LIMA graft. As both SVG grafts were known to be occluded, there was no need to spare their origins. Based on the life-threatening unstable condition, a lack of a surgical options, and the apparent feasibility of the endograft deployment, an urgent endovascular invasive approach was selected.

## Therapeutic intervention

**Figure 2:**
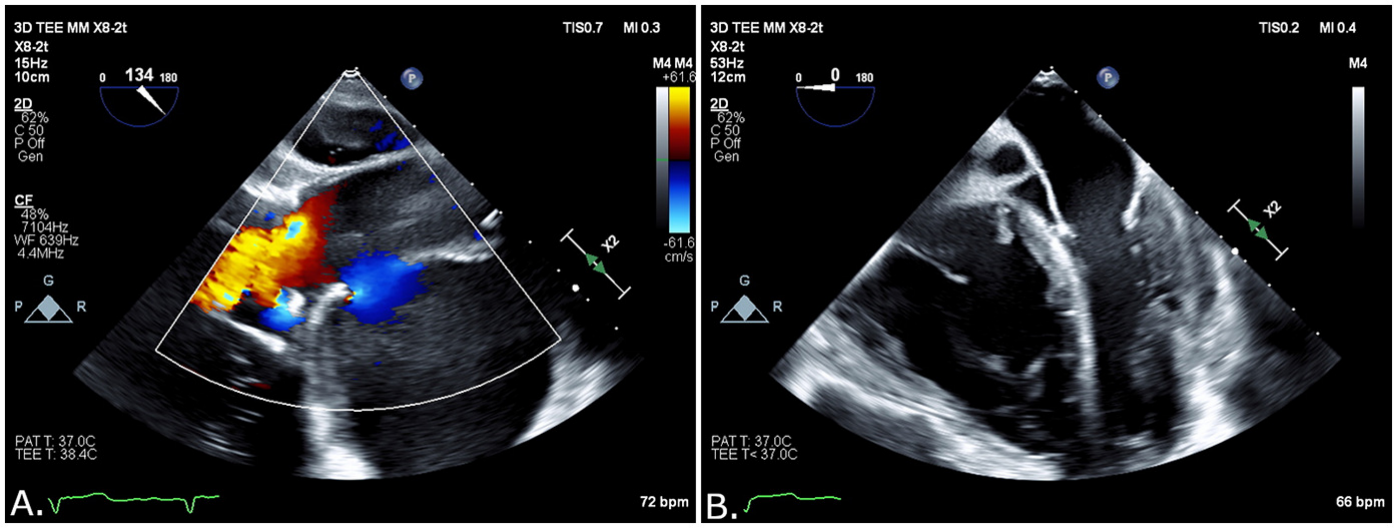
**(A)** Blood flow into the pseudoaneurysm opening by TEE. **(B)** Liquid and solid content on TEE.

The patient was immediately transferred to the catheterization laboratory. Access to the left femoral artery with an 8-F introducer for stent delivery and to the right femoral vein with a 6-F introducer for a temporary pacemaker was achieved under ultrasound guidance. Left radial access was used to place a pigtail catheter in the sinotubular junction (STJ). The procedure was performed under transesophageal echocardiography (TEE) imaging, which revealed a pseudoaneurysm with a mixed echo-dense and liquid content (Figures 2A,B and Supplementary Video S1) compressing the left atrium, resulting in a restrictive filling pattern.

An Amplatz extra stiff guide wire (Cook Medical, Bloomington, IN, USA) was advanced to the left ventricle. Standard stent graft GORE TAG 40/100 mm (W. L. Gore & Associates, Newark, DE, USA) was placed from the STJ toward the innominate artery. TEE and angiography confirmed optimal positioning without aortic valve compromise before deployment (Supplementary Video S2). Deployment was performed with rapid pacing to ensure minimal movement. Stent graft sealing of the pseudoaneurysm opening was then confirmed by angiography (Supplementary Video S3) and TEE (Supplementary Video S4). Improvement in left ventricular systolic function and hemodynamic parameters was noticed shortly after deployment. The patient was returned to the intensive cardiac care unit for further management.

**Figure 3:**
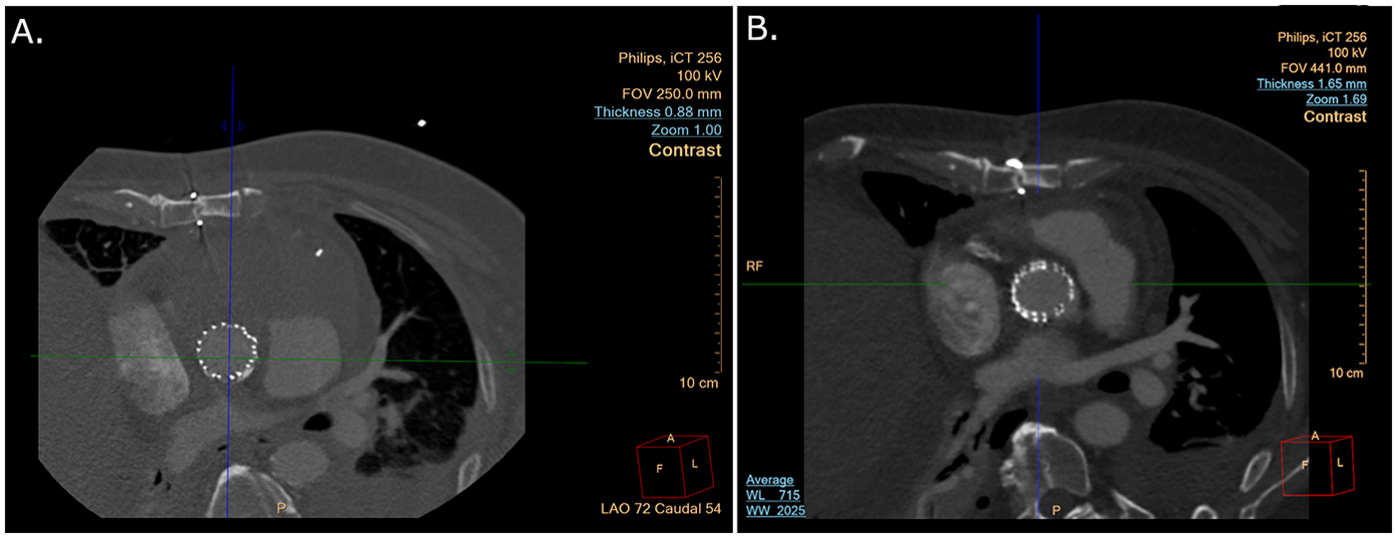
**(A)** CTA scan after endovascular pseudoaneurysm repair showing a large anterior mass. **(B)** CTA scan after endovascular pseudoaneurysm repair and hemisternotomy to remove blood clot and patch the aorta. CTA, computed tomography angiography.

Continued improvement allowed extubation a few hours later. Postoperative complications included right transudative pleural effusion, which required placement of a chest tube, and acute kidney injury with oliguria and creatinine up to 2.7 mg/dl. During the following days, kidney function rapidly improved back to baseline, allowing the performance of triphasic CTA. This demonstrated no endoleak but showed a large hematoma pressing on the heart in the anterior mediastinum (Figure 3A). Repeated TEE still showed restrictive cardiac physiology.

**Figure 4:**
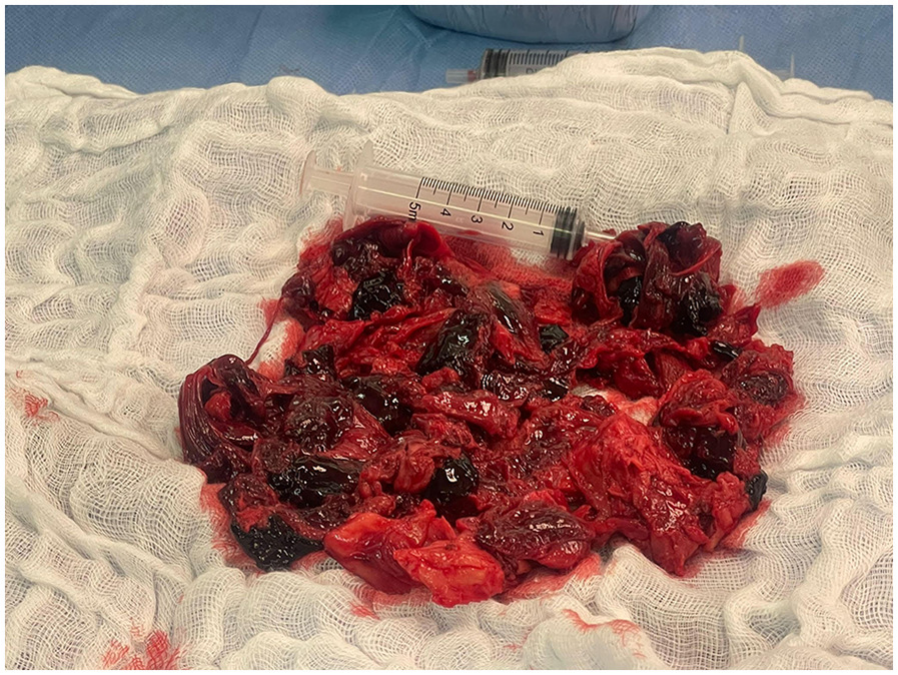
Cleared clots.

A second multidisciplinary heart team evaluation concluded that further intervention is needed to relieve the mediastinal pressure created by the remaining hematoma. As the patient was now stable to allow surgical intervention, a limited upper hemisternotomy (third intercostal space) was performed. The anterior mediastinum was cleared of blood clots (Figure 4) and the ascending aorta was exposed. The surgical team examined the stent graft confirming its position approximately 2 cm above the STJ. A small bleeding source was noticed from the lower end of the aortic opening. A bovine pericardial patch was mounted with bio-glue and manual pressure was placed on the patch for 5 min to ensure adequate patch alignment. Hemodynamic improvement was demonstrated, with rapid postoperative decline in central venous pressure from 23 to 13 mmHg. It should be noted that cultures from clots removed during the surgery and blood cultures taken during hospitalization were all negative.

The patient was extubated a few hours later, and chest tubes were removed. Follow-up CTA showed a reduced mediastinal mass with no apparent contrast extravasation from the covered stent (Figure 3B). The patient was discharged home 3 weeks after admission with preserved left and right ventricular function and a return to baseline kidney function. The patient was discharged with supplemental oxygen therapy due to atelectasis. The patient returned for routine follow-up visits at 2, 6, and 12 months postoperatively. He is currently feeling well and continues follow-ups through his primary care physician.

## Discussion

Traditional CABG surgery complications include bleeding, prolonged ventilation, infections, stroke, and vascular complications (16). It is not clear whether our patient developed the pseudoaneurysm because of the initial mediastinitis, a mechanical complication related to the CABG surgery, endogenous weakness of the tissue or some other cause. Post-CABG mediastinitis occurs in approximately 1% of patients (17) and is associated with worse short- and long-term outcomes (18). In our case, antibiotic treatment was administered but not completed due to patient non-compliance. Based on the 7 months between the development of mediastinitis and the initial aortic dilatation observed on TEE (Supplementary Figure S1), along with negative cultures from blood, bone fragments, and clots removed during surgery, we cannot confirm whether the initial mediastinitis caused the development of the pseudoaneurysm. Interestingly, the pseudoaneurysm origin and the location of the cardioplegia canula used in the CABG operation were very close, suggesting that iatrogenic trauma may be the cause, but confirmation is impossible.

To date, the gold standard treatment for ascending aortic pathologies requires surgical repair (1). However, surgical options carry a significant perioperative mortality in the range of 10%–20% (5) and are even prohibited by very high surgical risk in up to 20% of patients (5, 15). Percutaneous endovascular repair has matured and emerged as the first-line therapy for abdominal aortic pathologies. It therefore seems reasonable to attempt this treatment for patients with ascending aorta pathologies who are not eligible for surgical repair. The relatively small size of the ascending aorta, combined with its asymmetrical curvatures, results in geometric challenges for stent design. These are further complicated by the absolute need to avoid occluding the coronary arteries and allowing proper aortic valve function on one side while sparing the innominate and carotid arteries on the other side. In patients after CABG, further consideration of graft origins is of utmost importance. The first endovascular treatment of an ascending aortic dissection case was reported by Dorros et al. (19), with the first pseudoaneurysm reported by Lin et al. (20). Previous reports have shown promising results, with very high deployment success rates and a relatively low mortality rate of up to 14% (15, 21, 22). The prospective, multicenter, non-randomized, single-arm ARISE study of 19 patients with type A aortic dissection reported a 100% deployment success rate with two cases converting to open repair, three cases with observed endoleak (two at the time of the procedure and one 30 days later) and no case of device migration (23). As for adverse events, the authors reported a 15.8% all-cause mortality rate and 23.8% major adverse cardiovascular and cerebrovascular events (MACCE) at 30 days, with a 5.3% risk for major stroke. At the 12-month follow-up, 52.9% experienced an adverse event, with the majority of events being the need for “other surgeries/treatment/procedure” (23). A recent systematic review of retrospective cases and case series specific to non-dissection ascending aorta endovascular repair (aneurysms, pseudoaneurysms, penetrating aortic ulcers, intramural hematomas confined to the ascending aorta, thrombosis, iatrogenic coarctation, and aortic rupture) identified 105 cases, of which 71 (68%) were pseudoaneurysms. In this report, the in-hospital mortality rate was 2.9%, and at 8.9 months, all-cause mortality was 4.7%. In total, 21.9% of patients experienced complications, including endoleaks (10%) and stroke (5.7%) (8). For long-term outcomes, further data are needed; however, reports indicate a 75% 5-year survival rate (24) and a case of survival up to 8 years (25) after endovascular repair. In comparison, surgical repair carries a 16% in-hospital all-cause mortality rate (5) and a 13% risk of stroke (26). It should be noted that 50% of patients undergoing surgery were unstable at the time of surgery, with an in-hospital mortality rate of 31.4% compared to 16% in stable patients (26).

Detailed evaluation of the aortic anatomy is crucial before considering similar endovascular interventions. The ARISE study required deployment ≥2 cm distal to the most distal coronary artery ostia, with ascending aorta true lumen and total aortic diameters to be in the range of 24–42 mm. Trial screening identified 41 patients (19 in the main analysis and 22 for compassionate use), while 100 patients were excluded, primarily due to a short proximal landing zone or incompatible aortic diameters. Patients with aortic pathologies from connective tissue disease or active infections were also excluded due to increased risk. These criteria make endovascular repair feasible for only approximately 40% of non-surgical patients. Long-term event-free survival data are still needed to determine whether this strategy is best used as a bridge to surgery or as a destination therapy.

The upcoming ARISE II (NCT05800743) trial, which aims to recruit 370 high-risk patients with non-acute type A aortic pathologies, is expected to provide further insight into both the feasibility and outcomes of percutaneous endovascular repair of the ascending aorta.

This case presents further evidence supporting the use of endovascular ascending aorta repair in a non-surgical patient. In our patient, endovascular repair resulted in stabilization and sufficient improvement for the surgical removal of mediastinal clots and sealing of residual bleeding.

From the patient's perspective, percutaneous endovascular repair provided the only solution for a life-threatening condition. In addition, the minimally invasive procedure allowed for fast recovery.

Taken together, the current data seem to support the use of endovascular repair of the ascending aorta in both dissection and non-dissection pathologies in anatomically suitable patients. Further studies comparing surgical and endovascular options are warranted to determine accurate patient selection.

## Conclusions

Ascending aorta pseudoaneurysms are rare and potentially life-threatening. Surgical treatment must be performed promptly, as patient deterioration and instability can shift the risk-benefit balance unfavorably. A percutaneous endovascular approach may provide an urgent alternative for patients unsuitable for surgery. Further studies are warranted to determine the long-term benefits of such procedures.

## Data Availability

The original contributions presented in the study are included in the article/Supplementary Material, further inquiries can be directed to the corresponding author.

## Supplementary material

Supplementary Figure 1 Patient timeline.

Supplementary Video 1 TEE showing the ascending aorta with flow towards the pseudoaneurysm.

Supplementary Video 2 Angiography of the ascending aorta revealing contrast material flow into the pseudoaneurysm before intervention.

Supplementary Video 3 Angiography of the ascending aorta after applying the stent graft showing no leak into the pseudoaneurysm.

Supplementary Video 4 TEE post-procedural showing the normal aortic valve opening in close proximity to the stent graft.
