# Supplementary figures and images for: Case Report: Ascending hope—urgent endovascular repair of aortic pseudoaneurysm
Source: Front Cardiovasc Med. 2025 Sep 3;12:1532920. doi: 10.3389/fcvm.2025.1532920 (PMC12440952; doi:10.3389/fcvm.2025.1532920)

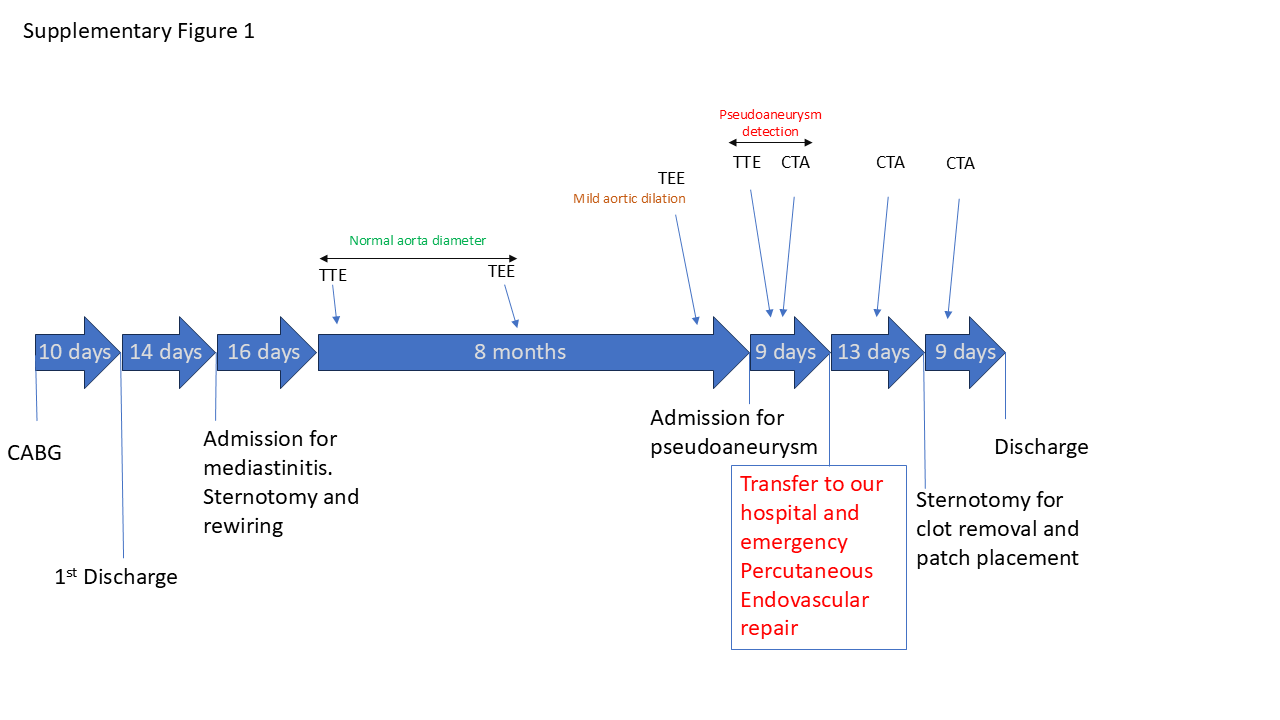

Supplement: Supplementary Figure 1 — Patient timeline. [file Image1.tif]
